# Functional Analysis of Hsp70 Inhibitors

**DOI:** 10.1371/journal.pone.0078443

**Published:** 2013-11-12

**Authors:** Rainer Schlecht, Sebastian R. Scholz, Heike Dahmen, Ansgar Wegener, Christian Sirrenberg, Djordje Musil, Joerg Bomke, Hans-Michael Eggenweiler, Matthias P. Mayer, Bernd Bukau

**Affiliations:** 1 Zentrum für Molekulare Biologie der Universität Heidelberg (ZMBH), DKFZ-ZMBH-Alliance, Heidelberg, Germany; 2 Deutsches Krebsforschungszentrum (DKFZ), Heidelberg, Germany; 3 Merck Serono, Global Research and Development, Darmstadt, Germany; University of South Florida Alzheimer's Institute, United States of America

## Abstract

The molecular chaperones of the Hsp70 family have been recognized as targets for anti-cancer therapy. Since several paralogs of Hsp70 proteins exist in cytosol, endoplasmic reticulum and mitochondria, we investigated which isoform needs to be down-regulated for reducing viability of cancer cells. For two recently identified small molecule inhibitors, VER-155008 and 2-phenylethynesulfonamide (PES), which are proposed to target different sites in Hsp70s, we analyzed the molecular mode of action in vitro. We found that for significant reduction of viability of cancer cells simultaneous knockdown of heat-inducible Hsp70 (HSPA1) and constitutive Hsc70 (HSPA8) is necessary. The compound VER-155008, which binds to the nucleotide binding site of Hsp70, arrests the nucleotide binding domain (NBD) in a half-open conformation and thereby acts as ATP-competitive inhibitor that prevents allosteric control between NBD and substrate binding domain (SBD). Compound PES interacts with the SBD of Hsp70 in an unspecific, detergent-like fashion, under the conditions tested. None of the two inhibitors investigated was isoform-specific.

## Introduction

The ubiquitous and highly conserved molecular chaperones of the 70 kDa heat shock protein (Hsp70) family are key players in protein homeostasis not only during stressful, but also optimal growth conditions. Members of the Hsp70 family are involved in folding of newly synthesized and misfolded proteins, solubilization of protein aggregates, degradation via the proteasome and autophagy pathways, transport of proteins through membranes, and assembly and disassembly of protein complexes [1]. Additionally, they are implicated in regulatory processes, involving the interaction with clients of the Hsp90 system [2], regulation of the heat shock response both in prokaryotes and eukaryotes [3], [4] and regulation of apoptosis [5]. Not surprisingly, Hsp70 chaperones have therefore been linked to numerous diseases, in particular folding disorders like Alzheimer's disease or Corea Huntington and many types of cancer [6].

All different functions of Hsp70s are achieved by a transient interaction of the chaperone with substrate proteins via its C-terminal substrate binding domain (SBD) [7]. This interaction is allosterically controlled by the nucleotide bound to the N-terminal nucleotide binding domain (NBD). In the nucleotide-free and ADP bound state the affinity for substrates is high but substrate association and dissociation rates are low. ATP binding to the NBD increases association and dissociation rates by orders of magnitude, thereby decreasing the affinity for substrates by 10- to 400-fold [8]–[10]. The Hsp70 cycle is in addition controlled by the action of co-chaperones, including J-domain proteins and nucleotide exchange factors. J-domain proteins in synergism with substrates stimulate the low intrinsic ATPase activity of Hsp70 and, thereby, facilitate efficient substrate trapping. Nucleotide exchange factors accelerate the release of ADP and subsequent ATP-binding triggers substrate release.

All eukaryotic cells contain several Hsp70 isoforms. In mammalian cells the most important Hsp70s are the constitutively, highly expressed cytosolic Hsc70 (HSPA8) and the heat-inducible cytosolic Hsp70 (HSPA1A, HSPA1B), the endoplasmic reticulum resident BiP (HSPA5) and the mitochondrial mortalin (HSPA9). Cancer cells seem to depend on high Hsp70 activity, possibly to buffer the effect of destabilizing mutations accumulating during cell immortalization and to counter the stress conditions resulting from the nutrient depleted, hypoxic microenvironment of the tumor. Thus, levels of the heat-inducible Hsp70 are increased drastically in a variety of human tumors and this observation often correlates with poor prognosis [11]. Furthermore, inhibition of Hsp90, which is currently being pursued actively as anti-cancer therapy and already in clinical trials, induces the heat shock response [12]. The resulting increase of Hsp70 levels is being made responsible for cancer cell survival and the relatively small therapeutic window of Hsp90 inhibitors. Therefore, the inhibition of Hsp70, either alone or in combination with Hsp90, is believed to be a promising path in anti-tumor therapy [13]. Such a strategy imposes important questions: Is it sufficient to inhibit only the heat-inducible Hsp70 for an effective anti-tumor therapy? What are the target structures and possible mechanisms of Hsp70 inhibition? Is it possible to find an inhibitor that is Hsp70 specific, not affecting the essential Hsc70 and BiP, given the high conservation within the Hsp70 family?

Whether targeting only the heat-inducible isoform is sufficient for successful anti-tumor therapy is currently debated. Depletion of Hsp70 using antisense RNA against HSPA1A/HSPA1B mRNAs induced apoptosis in several cancer cell lines but not in non-malignant cells [14]. In a different study reducing the levels of the heat-inducible Hsp70 had no effect and depletion of both Hsp70 and Hsc70 was necessary to reduce cell viability significantly [15]. Here we used siRNA to down-regulate different Hsp70 isoforms in cancer cells to reevaluate this question.

Based on the structure of Hsp70 proteins two potential inhibitor binding sites are apparent: the ATP binding pocket and the peptide binding cleft. The ATP binding pocket was considered to be a poor inhibitor binding site due to the mostly hydrophilic and electrostatic interactions with the ribose and phosphate moieties of the nucleotide [16]. In addition, the high conservation of the nucleotide binding site may prevent targeting of a specific Hsp70 paralog. Contacts of Hsp70 with polypeptides are dominated by hydrophobic interactions with several substrate residues, one of which inserts into a hydrophobic pocket of the SBD. In addition, a number of hydrogen bonds are formed between the peptide backbone of the substrate and the substrate-interacting loops of the SBD. Mimicking such complex interactions with a non-peptide small molecule appears to be difficult. However, sequence identity among Hsp70s is lower in the SBD and paralog-specific inhibitors appear feasible. Since the functional cycle of Hsp70s requires the mutual allosteric control of NBD and SBD [17], [18] and thus specific contacts between two domains, their docking-site could also be a potential drug binding site. However, for rational design of such an inhibitor structural information became available only recently [19]–[21]. Furthermore, Hsp70s interact with co-chaperones of the J-domain protein family and with nucleotide exchange factors and these interactions are essential for the chaperone activity of Hsp70s [22]. The corresponding interaction surfaces may serve as drug binding sites as well.

In recent years, several systematic attempts have been undertaken to identify small molecule inhibitors of Hsp70. A colorimetric unbiased screen identified several modulators of Hsp70 ATPase activity, which also influence protein folding [23]. A different study utilized a structure-based approach starting from adenosine to identify substances which would bind to the ATP binding pocket of Hsc70 [24]. The identified inhibitor VER-155008 binds Hsc70 with a dissociation equilibrium constant (*K_d_*) of 0.3 µM and inhibits tumor cell growth with a GI_50_ of 5 to 14 µM. Recently, 2-phenylethynesulfonamide (PES, also known as pifithrin-μ), which acts as an inhibitor of the mitochondrial branch of p53-mediated apoptosis [25], was reported to bind specifically to and inhibit the protein-folding activity of Hsp70 [26]. The mode of action remained enigmatic, but it was proposed that only the heat-inducible Hsp70, not the constitutively expressed Hsc70, interacts with PES and that this interaction is mediated by the C-terminal SBD. A more recent study relativized these findings and suggests that PES does not discriminate between Hsp70 and Hsc70 [27].

To explore the full potential and elucidate the molecular mechanism of two drug candidates, which presumably target different structures in Hsp70 and Hsc70, respectively, we examined the isoform specificity of VER-155008 and PES and the effect of these inhibitors on individual steps of Hsp70's functional cycle, including nucleotide binding, ATP hydrolysis, substrate interaction and interdomain communication. This analysis revealed new insights into the mode of action of Hsp70 inhibitors and point out some pitfalls in Hsp70-centered drug design.

## Materials and Methods

### Proteins and Inhibitors

Human Hsp70, Hsp70(1–382) and Hsc70 were purified as His_6_-Sumo fusions essentially as described [28]. For some experiments, C-terminally FLAG-tagged Hsp70 was used. This variant behaved in all biochemical assays like untagged Hsp70. Nucleotides were removed a previously published [29]. To allow binding to Resource Q the salt concentration was lowered to 10 mM KCl. Hdj1 and Apg2 were cloned and purified as a His_6_-Sumo fusion as published [30], [31] using buffer LBW150 (40 mM Hepes-KOH pH 7.6, 150 mM KCl, 5 mM MgCl_2_, 5% (w/v) glycerol, 10 mM β-mercaptoethanol) and Protino Ni-IDA material (Macherey-Nagel). Firefly luciferase was purified as described [32]. Pyruvate kinase/lactate dehydrogenase (PK/LDH) enzyme mix was obtained from Sigma.

PES (pifithrin-μ) was purchased from Sigma. VER-155008 was synthesized according to a published procedure [24]. Inhibitors were dissolved in dimethyl sulfoxide (DMSO) and stored at −20°C.

### siRNA mediated knockdown and viability assay

Cell lines BT474, HT29, HCT116, PC3, SKBr3 as well as MCF7 were obtained from ATCC; A2780 were obtained from ECAAC and MDA-MB-468 from DSMZ. Passages stored in the central cell bank at Merck Serono were tested for their identity by using STR (short tandem repeat) analysis. Cell lines were usually passaged for maximal 3–4 month and only passage number <30 are used for the shown experiments. To perform specific knockdown of the single heat shock protein family members siRNA transfection on breast cancer cell line MDA-MB-468 was carried out using Lipofectamine 2000 (Invitrogen) in 96 well format with a siRNA concentration of 24 nM and six replicates per transfection. Single or pooled siRNAs specific for indicated HSPA proteins were used. As controls siGenome non-targeting siRNAs (Dharmacon) or transfection medium alone (TM) were utilized. To confirm successful transfection cells were transfected with siRNAs against the mitotic kinase PLK1 (siGenome Smartpool, Dharmacon), which induce a mitotic arrest. Controls were put on each 96 well plate. All siRNAs were purchased from Dharmacon or Qiagen.

Knockdown efficiency was checked by Western blot analysis. For this purpose proteins were extracted by using RIPA lysis buffer (10 mM Tris/HCl pH 7,4, 150 mM NaCl, 1% NP40, 1% Triton X100, 0,4% Na-Deoxycholat, 2 mM EDTA, 0,3% SDS+protease inhibitors [Complete, Roche]). 6 µg total protein was resolved on SDS PAGE (Criterion, 4–20% SDS gel, Biorad) and transferred to PVDF membrane (Immobilon FL, Millipore). Western blot staining was done with HSPA1 and HSPA8 specific antibodies from Stressgen (SPA-810 and SPA-815) and infrared-labeled secondary antibodies (donkey anti mouse IRDye800 [Rockland] and goat anti rat Alexa680 [Molecular Probes]). Knockdown efficiency was quantified by detecting signals with Odyssey infrared imaging system.

Viability of cell lines after knockdown of heat shock proteins was measured by using Alamar blue reagent (Serotec) according to the manufacturer's manual. Averages of all replicates were calculated and viability was specified as % of TM

### Chaperone activity assays

Refolding of thermally denatured firefly luciferase was performed as published [33] with a modified refolding buffer (25 mM Hepes-KOH pH 7.5, 10 mM potassium acetate, 5 mM magnesium acetate, 2.5 mM DTT). Inhibitors or DMSO were added after denaturation to the final concentrations indicated.

### ATPase activity determination

Steady-state ATPase rates were determined photometrically with a coupled assay. 4 µM Hsp70 or Hsc70 were assayed in the absence and presence of 1 µM Hdj1 and the indicated concentrations of inhibitors. The reaction mixture additionally contained 1 mM phosphoenol pyruvate, 0.25 mM NADH and 1/100 vol. PK/LDH mix in refolding buffer. The reaction was started by the addition of ATP with a range of concentrations between 0.2 and 200 µM and the decrease of absorption at 340 nm was followed for 60 min in a microplate reader (FLUOstar Omega, BMG Labtech). Michaelis-Menten constants (*K_M_*) and dissociation equilibrium constants of the inhibitor (*K_i_*) were obtained by global fitting of the data.

### Kinetic measurements

For measuring ADP association rates nucleotide-free Hsp70 (2 µM) was mixed with 1 µM N8-(4N′-methylanthraniloylaminobutyl)-8 aminoadenosine 5′-triphosphate (MABA-ADP, [29]) in a stopped-flow apparatus (SX.18MV, Applied Photophysics; λ_ex_ 360 nm, cut-off filter 420 nm) to follow changes in fluorescence intensity. We fitted the equation for two or three phase association to the data to obtain apparent association rates.

Peptide dissociation equilibrium and rate constants as well as apparent association rates were determined as published [10], [28], [34] using d-NR-peptide (dansyl chloride-labeled NR-peptide (NRLLLTGC), Peptide Specialty Laboratories GmbH). For the determination of K_d_ and dissociation rate constant (k_off_), nucleotide-free Hsp70 was incubated with d-NR peptide for at least 30 min at 30°C before measurements.

### Isothermal Titration Calorimetry (ITC)

ITC measurements were performed with a VP-ITC micro calorimeter (Microcal, LLC/GE Healthcare Bio-Sciences AB, Uppsala, Sweden). ITC data analysis was performed using Origin 7 calorimetry software from the same supplier. The titration buffer used was 30 mM HEPES pH 7.5; 2 mM Na-phosphate; 1 mM MgCl_2_; 1 mM TCEP; 100 mM KCl. The proteins were conditioned into the titration buffer before titrations by spin filtration using Zeba Desalt Spin Column (Pierce) that were equilibrated in the same buffer. All titrations were performed in regular direction of titrating the respective ligands to the target protein with 12 µl injections. If not otherwise indicated the concentrations were 60–100 µM for the ligands (syringe) and 4–10 µM for the proteins (cell) with a maximum final concentration of 1% DMSO in both compartments. The HSP70-proteins were all nucleotide free. In order to investigate ligand interactions of PES in presence of nucleotide the titration buffer was complemented with 100 µM ADP in both compartments. All binding experiments were carried out at 30°C.

### Differential scanning calorimetry

DSC measurements were performed with an automated VPDSC capillary cell micro calorimeter (Microcal, LLC/GE Healthcare Bio-Sciences AB, Uppsala, Sweden). The buffer and the protein conditioning was the same as described for ITC experiments. The final concentration of HSP70 variants was 7.5 µM and for ligands 100 µM with exception of VER-155008 that was used at 50 µM. DMSO was included in all samples at 1% final concentration. DSC data analysis was performed using Origin 7 calorimetry software from the same supplier. After concentration normalization and baseline correction, thermograms could be fit to a non 2-state unfolding model with two overlaying transitions. In order to facilitate the judgement on the relative binding interaction we report the apparent T_m_ value taken at the peak maximum.

### Surface plasmon resonance (SPR) spectroscopy

Full-length Hsp70 was immobilized onto CM5 (series S) sensor chips using standard amine coupling at pH 6.0. Hsp70 inhibitor compounds were diluted in running buffer (20 mM HEPES, 150 mM KCl, 2 mM MgCl_2_, 1 mM DTT, 0.1 mM EDTA, 0.03% Tween20, 2% DMSO pH 7.4) and analyzed with a Biacore S51 (Biacore AB, GE Healthcare Life Sciences, Uppsala, Sweden) using a 2-fold dilution series. The highest compound concentration varied according to the expected dissociation constant, but all compounds were tested at 10 different concentrations. Interaction analysis cycles were run at 30 µL/min and consisted of a 120 s sample injection followed by 180 s of buffer flow (dissociation phase). All sensorgrams were evaluated by first subtracting the binding response recorded from the control surface (reference spot), followed by subtracting a buffer blank injection. To determine kinetic rate constants, data sets were fitted to a simple 1∶1 interaction model including a term for mass transport using numerical integration and nonlinear curve fitting.

### Crystallization and structure refinement

The nucleotide binding domain of human Hsp70 (residues 1–382) was crystallized at 4°C by hanging drop vapour diffusion against 0.1 *M* Tris, 22% PEG 10,000, 0.1 M NaCl, 20% glycerol, 2% DMSO, 2 mM VER-155008, pH 9.0. The crystals had dimensions of 0.2×0.2×0.3 mm^3^ and belonged to the monoclinic space group P2_1_. X-ray diffraction data to a 2.6 Å resolution were collected on X06SA-PX beamline at Swiss Light Source (SLS) synchrotron radiation source using a Pilatus detector [35] and the images were indexed, integrated and scaled using XDS program package [36]. The data were collected using a cryo-cooled crystal at 90 K in the crystallization buffer.

The structure was solved by molecular replacement using the program MOLREP from the CCP4 program suite [37]. The structure 1S3X from the Protein Data Bank (PDB) was used as the search model. Subsequently, several cycles of refinement using the program Buster (vers. 2.11.1., Global Phasing Ltd., Cambridge, UK) and crystallographic model building using the graphic package COOT [38] were applied. The resulting final model had R_work_/R_free_ of 0.167/0.239.

## Results

### Simultaneous reduction of Hsp70 and Hsc70 levels is required to compromise cancer cell viability

**Figure 1:**
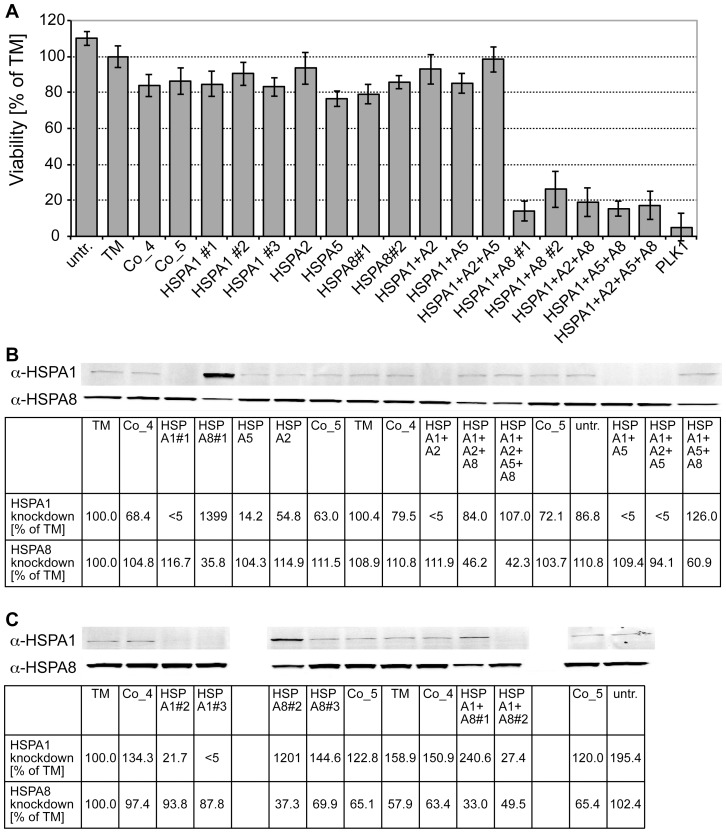
Simultaneous knockdown of HSPA1 and HSPA8 are necessary to compromise viability of MDA-MB-468 breast cancer cells. MDA-MB-468 cells were transfected with siRNAs targeting the indicated heat shock proteins. For HSPA1 as well as HSPA8 and HSPA1+HSPA8 several single siRNAs or mixes were used marked with #1–3. **A**, Cell viability was measured six days after transfection by using Alamar Blue reagent. As positive control a pool of siRNAs against the mitotic kinase PLK1 was transfected. As negative controls non-targeting siRNAs (Co_4 and Co_5) were used, additionally cells were incubated only with the transfection reagent without siRNAs (TM) or left untreated (untr.). Viability is shown as % of TM. Only simultaneous transfection of HSPA1 and HSPA8 specific siRNAs induced a significant loss of viability. Additional knockdown of HSPA2 or HSPA5 had no further effect. **B** and **C**, Knockdown was confirmed by Western Blot analysis using HSPA1 and/or HSPA8 specific antibodies and infrared-labeled secondary antibodies three days after transfection of siRNAs. Knockdown efficiency was quantified by detecting signals with Odyssey infrared imaging system and is specified as % of TM.

We first addressed the question which of the Hsp70 paralogs is required for viability of model cancer cells (MDA-MB-468 breast carcinoma cells). To specifically down-regulate the different Hsp70 paralogs we used siRNA technology. Three different siRNAs or siRNA pools each for HSPA1A/HSPA1B and HSPA8 and, in addition, siRNA pools against HSPA2, which is expressed in a tissue specific manner [39] and was implicated in cancer cell survival [40], and HSPA5 were utilized. Efficacy of siRNA against HSPA1 and HSPA8 was checked by immuno blot analysis using specific antisera (Figure 1). Individual siRNAs against any of the tested Hsp70s did not decrease cell viability significantly as compared to control siRNAs (Figure 1A). A combination of siRNAs against HSPA1, HSPA2 and HSPA5 also had no effect. A combination of siRNA against HSPA1 and HSPA8 was necessary to reduce cell viability. Similar results were obtained for colony formation and viability of other cancer cell lines (Table S1 and S2). The effectiveness of siRNA treatment was verified by Western blotting (Figure 1B and C). Two of the three siRNAs against HSPA1 reduced the protein levels to less than 10% of the untreated cells. As expected, down-regulation of HSPA8 induced the heat shock response and thus increased the level of Hsp70. We conclude that simultaneous reduction of the levels of constitutive Hsc70 and the inhibition of the thereby caused up-regulation of heat-inducible Hsp70 is necessary to compromise cell survival. Therefore, a paralog-specific Hsp70 inhibitor may not be appropriate for anti-cancer therapy.

### VER-155008 and PES inhibit Hsp70- and Hsc70-mediated luciferase refolding

**Figure 2:**
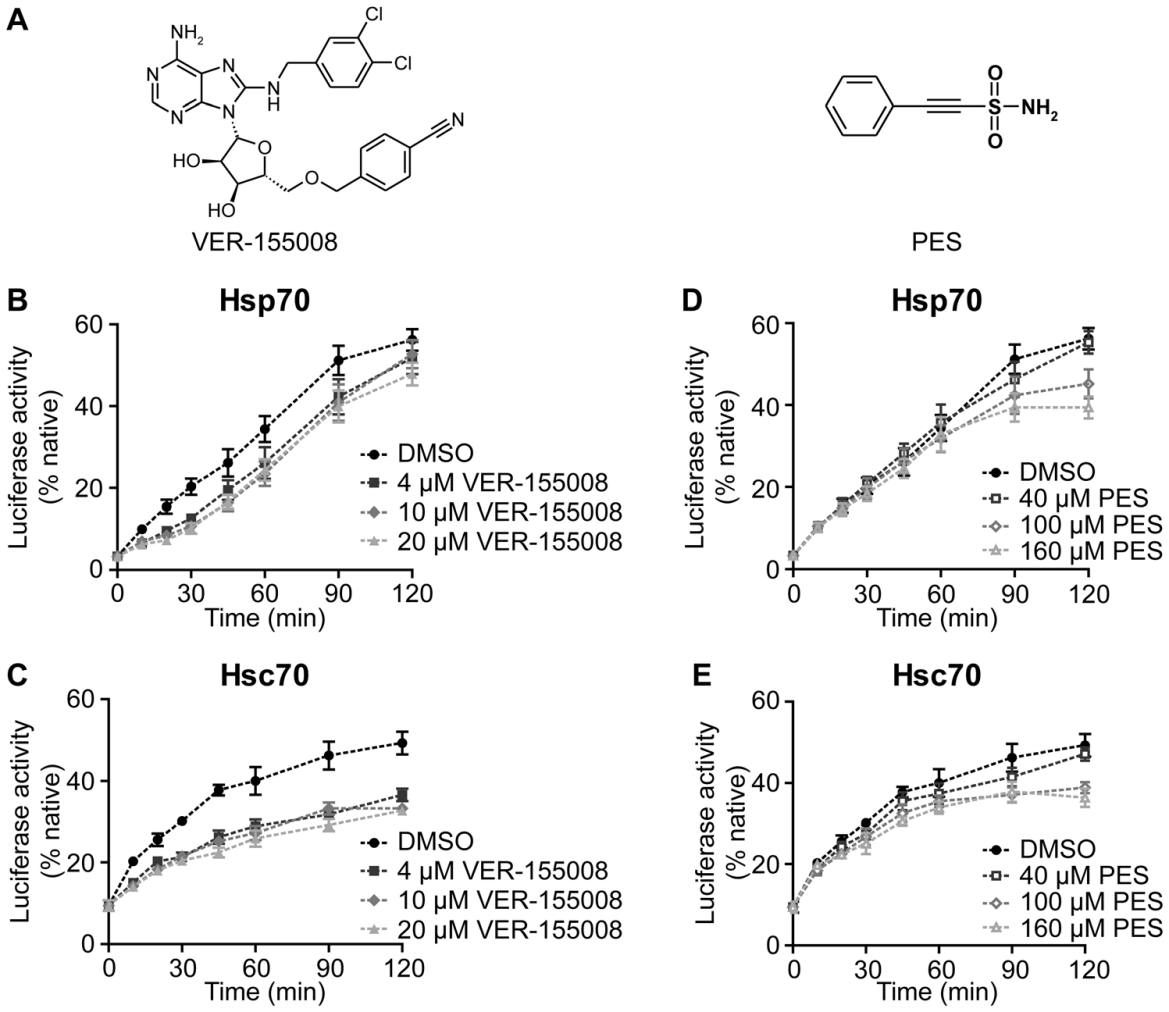
VER-155008 and PES inhibit reactivation of firefly luciferase. **A**, Structure of the small molecule inhibitors VER-155008 and PES. **B–E**, Refolding of thermally denatured firefly luciferase by Hsp70 (**B, D**) and Hsc70 (**C, E**) in the presence of different concentrations of VER-155008 (**B, C**) or PES (**D, E**) (80 nM luciferase, 4 µM Hsp70/Hsc70, 2 µM Hdj1, 0.4 µM Apg2). Luciferase activity is plotted as a fraction of the native luciferase control. Error bars represent standard errors of three independent experiments. ANOVA analysis of the 120-min-values indicates highly significant differences between control and inhibitor treated samples (p<0.001) except for Hsp70 inhibition by VER-155008 (p = 0.011).

While an inhibition of refolding of heat denatured firefly luciferase by VER-155008 (Figure 2A) in rabbit reticulocyte lysate was demonstrated earlier [41], the impact of this compound on a purified chaperone-based protein refolding system was not assessed. The influence of the compound PES (Figure 2A) on the refolding of a denatured protein by Hsp70 was also not investigated previously. For this reason and to verify isoform specificity of the two compounds, we performed luciferase refolding experiments in the presence of either Hsp70 or Hsc70, the J-domain co-chaperone Hdj1 (DNAJB1) and the nucleotide exchange factor Apg2. We found that in this purified system about half of the luciferase can be reactivated within 120 min by either Hsp70 or Hsc70 (Figure 2B–E). Interestingly, the reactivation kinetics show differences between Hsp70 and Hsc70, arguing for divergent interactions of the two chaperones with either substrate or co-chaperones. At 4 µM VER-155008 partially inhibited luciferase refolding by both Hsp70 and Hsc70 (Figure 2B, C). While the refolding yield of the Hsc70-mediated refolding was significantly reduced, the refolding reaction by Hsp70 was rather delayed and yields after 120 min were only slightly reduced (statistically not significant). Only 2.5 and 5-fold higher VER-155008 concentrations led to statistically significant inhibition (ANOVA p = 0.011). The reduction of luciferase reactivation caused by the presence of PES is smaller than for VER-155008 during the initial phase of refolding and only becomes apparent at later stages. Interestingly, in contrast to the original report [26] PES did not discriminate between Hsp70 and Hsc70 (Figure 2D, E).

### VER-155008 is a competitive inhibitor of the Hsp70 ATPase activity

**Figure 3:**
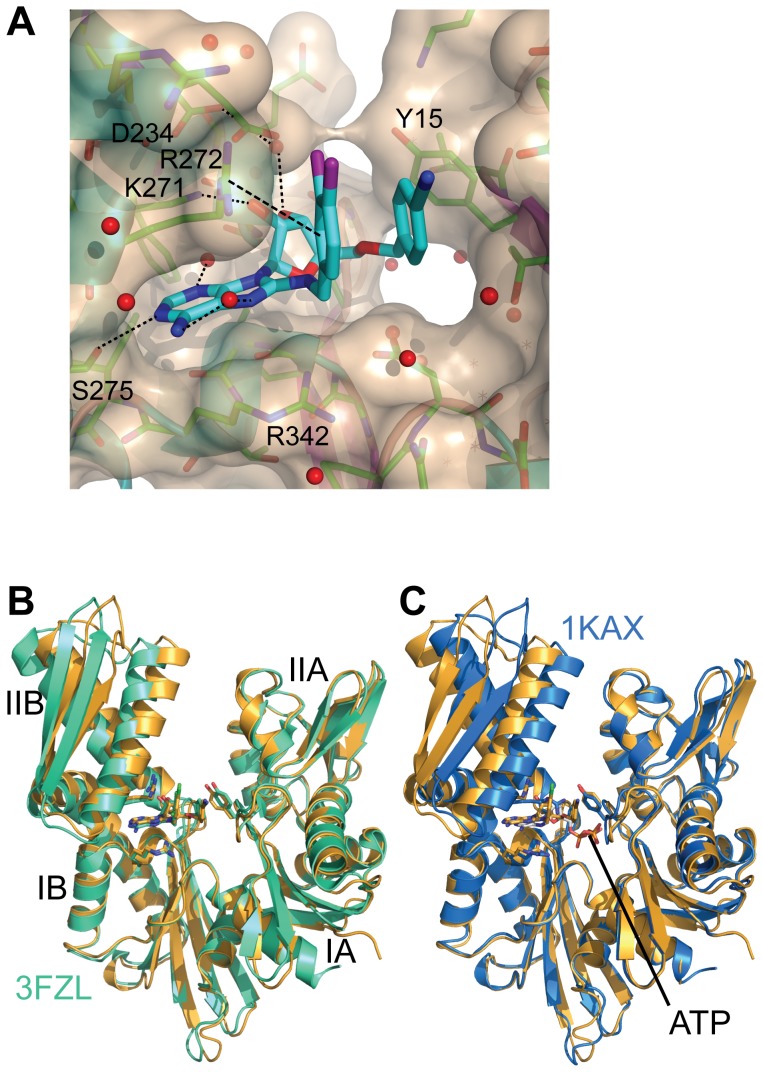
Crystal structure of human Hsp70 NBD in complex with VER-155008. (PDB entry code 4IO8). **A**, Zoom into the nucleotide binding pocket with NBD in cartoon and transparent surface representation. VER-155008 interacting residues are shown as sticks with hydrogen bonds (K271, S275 and water molecules) and π-stacking R272 indicated as dotted and dashed lines, respectively. **B**, overlay of the structure of human Hsc70 NBD in complex with Bag1 and VER-155008 (green, PDB ID 3FZL; [24]) and our structure (gold) in cartoon representation with VER-155008 in stick representation colored according to the elements with carbon colored in green (3FZL) and gold (our structure). **C**, overlay of bovine Hsc70 NBD K71M variant in complex with ATP (blue, PDB ID 1KAX; [58]) and our structure (gold) in cartoon representation with VER-155008 and ATP in stick representation colored according to the elements with carbon colored in blue (1KAX) and gold (our structure).

VER-155008 was previously shown to compete with ATP and ADP for binding to Hsp70 [24]. Moreover, a crystal structure of the compound in complex with Hsc70 and the Bag-domain of the nucleotide exchange factor Bag-1 clearly shows its interaction with the ATP binding pocket [24], [41]. To elucidate how VER-155008 affects the conformation of human Hsp70 in the absence of a nucleotide exchange factor we solved the crystal structure of the NBD of human Hsp70 in complex with VER-155008 (Figure 3; data collection and the refinement statistics can be found in Table S3). As expected, the adenine moiety of VER-155008 was inserted into the adenine binding pocket between Arg272 and Arg342 forming hydrogen bonds between O^γ^ of Ser275 and N^1^ of the adenine ring. O^2'^ of the ribose part of VER-155008 makes a direct hydrogen bonding contact to N^ζ^ of Lys271. In addition, O^3'^ of the ribose group forms another hydrogen bond to a water molecule, which is hydrogen-bonded to O^δ^ of Asp234. π-stacking between the side chain of Arg272, the dichlorobenzene and the 4-cyano-benzyloxymethyl moieties is similar to the one previously described [24]. Nevertheless, the angle of the 4-cyano-benzyloxymethyl ring of the inhibitor and between the side chain of Tyr15 is about 50°, which is out of the range that is usually described as π-π stacking interaction. Overall the structure is similar to the previously solved structure of VER-155008 in complex with Hsc70 NBD and the nucleotide exchange factor Bag1. However, the central cleft between lobe I and II is not as large due to the fact that subdomain IIB is rotated by 5 – 6° towards subdomain IB as compared to the previous structure (Figure 3B). The reason for this observation is most likely the absence of the nucleotide exchange factor Bag1, which is known to cause a 14° outward rotation of subdomain IIB [42]. As compared to the ATP bound structures of the Hsc70 NBD subdomain IIB is rotated outward by some 8° (Figure 3C), indicating that VER-155008 binding to Hsp70 does not allow the complete closure of the central cleft of the NBD.

**Figure 4:**
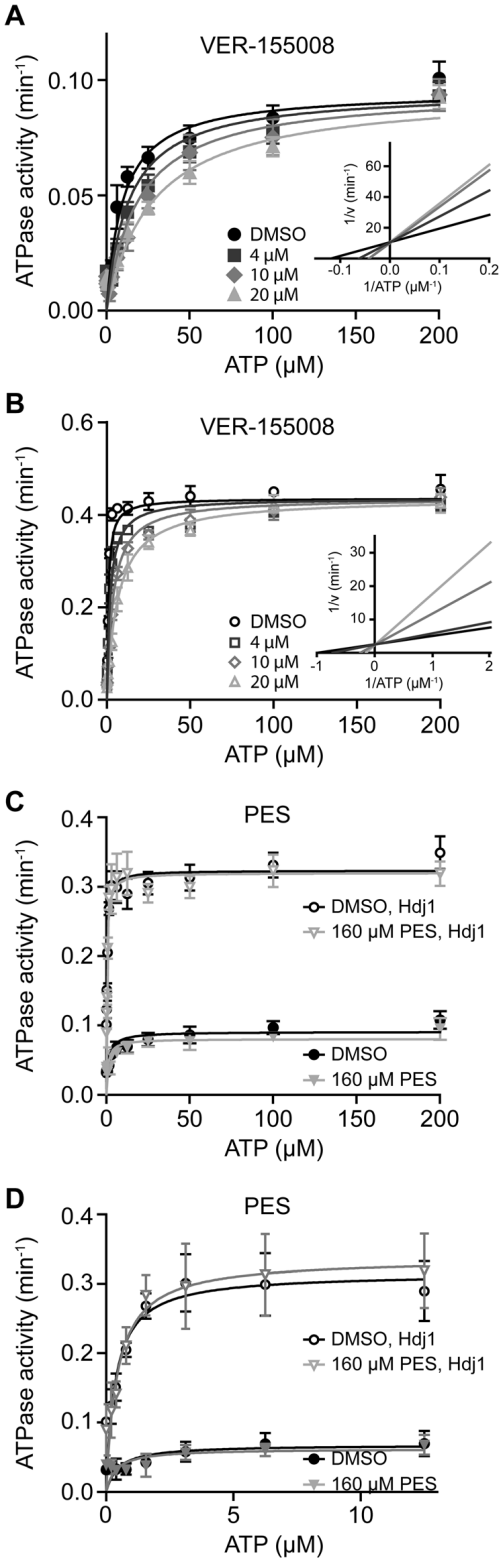
VER-155008 inhibits Hsp70's ATPase activity in a competitive manner and slows down nucleotide association. Steady-state ATPase activity of Hsp70 (4 µM) in the absence (**A, C**) or presence (**B, C**) of 1 µM Hdj1. VER-155008 (**A, B**) and PES (**C, D**) were added as indicated. **D**, zoom of **C** for 0 to 12 µM ATP. *K_M_* and *K_i_* values for VER-155008 were determined by global fitting of the data. Differences in K_M_ and k_cat_ in the presence of PES as compared to the DMSO control were not statistically significant (t-test, p values of 0.27 to 0.85). In contrast, differences in apparent K_M_ observed in the presence of VER-155008 was statistically significant (p = 0.05 in absence of Hdj1; p = 0.005 in presence of Hdj1). Error bars represent the standard errors of at least three independent measurements. **E, F,** Association of MABA-ADP (0.5 µM) to nucleotide-free Hsp70 (0.5 µM) in the presence of VER-155008 (20 µM) and PES (160 µM). Changes in fluorescence intensity were followed in a stopped flow device for 20 (**E**) and 500 seconds (**F**). Comparison of the ADP association rates in the absence and presence of PES using the students t-test resulted in a P value of 0.41, indicating that the differences are not statistically significant.

To further characterize the binding of VER-155008 to Hsp70 we performed steady-state ATPase assays to determine the effective dissociation constant of the inhibitor *K_i_* for Hsp70 in the absence and presence of Hdj1 (Figure 4A, B). A global fit of steady-state ATPase activities at different ATP- and inhibitor concentrations resulted in a *K_i_* value of 10.9±2.8 µM with a Michaelis constant (*K_M_*) of 9.9±1.3 µM and a maximum rate (*v_max_*) of 0.095±0.003 min^−1^ (Figure 4A). In the presence of the co-chaperone Hdj1 the maximum ATPase rate increased 4.5-fold to 0.436±0.005 min^−1^ and *K_i_* and *K_M_* were reduced to 2.87±0.39 µM and 0.837±0.081 µM, respectively (Figure 4B).

In contrast, PES, even at the high concentration of 160 µM, did not affect the intrinsic ATP hydrolysis rate of the chaperone. The stimulation of ATPase activity by Hdj1 also remained unchanged (Figure 4C, D). This observation is consistent with the hypothesis that PES interacts with Hsp70 through the substrate binding domain [26].

A competition between the inhibitor VER-155008 and nucleotide for binding to the same binding pocket should result in a decrease of the observed nucleotide association rate. To test this hypothesis we mixed nucleotide-free Hsp70 and the fluorescently labeled ADP derivative MABA-ADP in a stopped-flow device (Figure 4E). In the absence of inhibitors, we observed a biphasic association, consistent with a two-step mechanism with a fast formation of an encounter complex and a subsequent slower conformational change [29]. At our experimental conditions the first phase of ADP association (60% of total fluorescence change) occurred with a rate of 2.22±0.06 s^−1^, resulting in a half-life of 0.31 s (Table 1), and the second phase with 0.54±0.05 s^−1^. In the presence of PES the ratio between fast and slow association remained unchanged, as well as the apparent association rates with 2.09±0.14 s^−1^ and 0.49±0.05 s^−1^, respectively (Figure 4E). VER-155008, however, significantly slowed down the observed association of MABA-ADP to Hsp70 (Figure 4F). Nucleotide bound to Hsp70 in a triphasic reaction with rates of 0.26±0.02 s^−1^, 0.032±0.001 s^−1^, and 0.0075±0.0001 s^−1^. The lowest rate contributed 80% of the amplitude indicating that dissociation of the inhibitor, which is rate-limiting under these conditions, occurs mainly with a half-life of 92 s.

**Table 1:** Kinetic constants of the Hsp70-substrate and Hsp70-nucleotide interaction in the presence or absence of inhibitors.

	MABA-ADP		d-NR-peptide		
	association		*K*_d_ (µM)	association	*k*_off_
	(10^−3^ s^−1^)			(10^−3^ s^−1^)	(10^−3^ s^−1^)
	slow phase	fast phase			
DMSO	557±7	2255±44	4.0±0.3	6.8±0.02	3.35±0.21
VER-155008	7.8±0.22	49±10	n.d.	6.4±0.01	3.26±0.26
PES	445±12	1872±63	4.0±0.9	6.5±0.02	3.71±0.26

n.d., not determined.

### Inhibitors do not affect chaperone - substrate interactions

**Figure 5:**
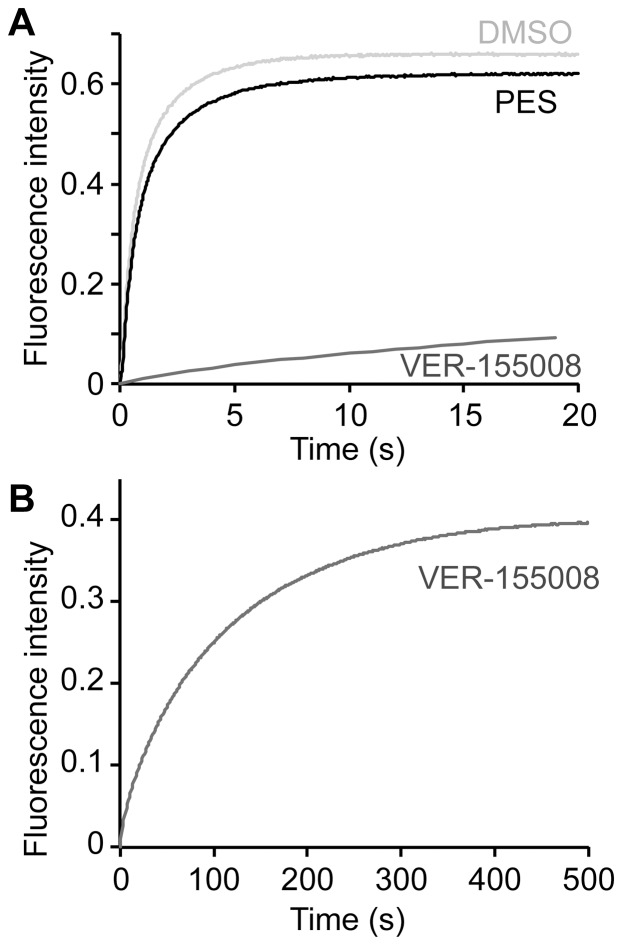
Substrate binding remains unchanged in the presence of small molecules. **A** and **B**, PES does not block the Hsp70-substrate interaction. Dissociation equilibrium titration of Hsp70 with d-NR-peptide. **A**, Selected fluorescence emission spectra of d-NR-peptide bound to different concentrations of Hsp70 as indicated in the presence or absence of 160 µM PES. **B**, Fluorescence intensity at 525 nm plotted against the Hsp70 concentration (one of four independent experiments shown). We determined the *K_d_* values by fitting the quadratic solution of the law of mass action to the data (Table 1). Paired t-test analysis did not reveal any statistically significant difference between the K_d_ values in the absence and presence of PES (p = 0.53). **C**, Dissociation of d-NR peptide from Hsp70 in the presence of VER-155008 or PES. The fluorescence decay was fitted by a single exponential decay to obtain *k_off_* values (Table 1). **D**, Fluorescence changes observed during the association of 1 µM d-NR-peptide to 1 µM Hsp70 in the absence and presence of inhibitors. ANOVA analysis demonstrated that neither VER-155008 nor PES caused any statistically significant difference (p = 0.32). **E and F**, ATP-stimulated dissociation of d-NR-peptide from Hsp70 in the presence of different concentrations of VER-155008 (**E**) and PES (**F**) as measured in a stopped flow apparatus. ANOVA analysis of the results show that the differences observed in the presence of VER-155008 are highly statistically significant (p<0.0001). In contrast, differences in the presence of PES are not significant (p = 0.63). The individual curves in **C–F** are y-transformed for better visibility.

Based on our findings and the report by Leu et al. [26] a possible mode of action of PES is an inhibition of substrate binding to the Hsp70 chaperones. To test this hypothesis we analyzed binding of a fluorescently labeled substrate peptide (d-NR, dansyl chloride labeled NRLLLTGC, [34]) to different concentrations of nucleotide-free Hsp70 under steady state conditions in the absence and presence of PES (Figure 5). The dansyl group attached to the peptide changes fluorescence intensity in response to the environment, with bound peptide showing a brighter signal. The observed increase in fluorescence with higher Hsp70 concentrations was comparable in the DMSO control and in the presence of 160 µM PES (Figure 5A). The determined *K_d_* values of 4.0±0.3 µM and 4.0±0.9 µM in the absence or presence of PES, respectively, clearly demonstrate that the inhibitor did not affect the affinity of Hsp70 for the peptide substrate (Figure 5B, Table 1).

Although substrate affinity was not altered in the presence of PES, the inhibitor might influence the dynamics of substrate association and dissociation. To test this hypothesis we analyzed the release of fluorescently labeled substrate peptide from Hsp70 in the high affinity state. As seen in Figure 5C the decrease in fluorescence intensity upon addition of unlabeled quench peptide was similar in the presence of PES and DMSO and the determined dissociation rate constants, (3.35±0.21)·10^−3^ s^−1^ and (3.71±0.26)·10^−3^ s^−1^, respectively, were not significantly different. With similar *K_d_* and *k_off_* values the association rate constants must remain unchanged in the presence of inhibitors. Therefore, we only analyzed binding of d-NR at a single concentration (Figure 5D, Table 1). The substrate indeed bound to Hsp70 with identical kinetics in the presence and absence of PES. These results clearly show that PES neither directly affects the affinity of Hsp70 for peptide substrates, nor the kinetics of binding and release, making an inhibitory mechanism mediated by the substrate binding domain unlikely.

### VER-155008 but not PES affects inter-domain coupling

The mutual allosteric regulation of NBD and SBD is an important feature of Hsp70 chaperones. While binding of substrates stimulates ATP hydrolysis, the rebinding of ATP to the nucleotide-free chaperone induces conformational changes in the SBD, which result in accelerated peptide release [9], [29], [43]. We used this feature to study the consequences of PES and VER-155008 binding for interdomain communication. First, we investigated the influence of VER-155008 binding to the NBD on peptide association and dissociation. As shown in Figure 5C and D, VER-155008 had no influence on substrate binding and release. Second, we analyzed the influence of VER-155008 and PES on ATP-stimulated substrate release. Nucleotide-free Hsp70 was loaded with d-NR-peptide in presence of different inhibitor concentrations and subsequently mixed with ATP and unlabeled NR-peptide in a stopped-flow apparatus. In the absence of VER-155008 (DMSO-control) the ATP-induced peptide dissociation rate was 4.30±0.05 s^−1^ consistent with published results for nucleotide-free Hsp70 [34]. The dissociation of the labeled peptide slowed down with increasing VER-155008 concentrations to reach a value of 0.53±0.01 s^−1^ at 10 µM VER-155008 (Figure 5E). This rate is similar to the rate of ATP-induced peptide release in the presence of ADP (0.43 s^−1^, [34]). This finding is consistent with VER-155008 and ATP competing for binding to the NBD. These data also show that VER-155008 does not trigger the conversion of Hsp70 into the low-affinity (ATP) state. PES, in contrast, did not influence the release rates at any concentration analyzed (Figure 5F) and therefore has no major effect on the allosteric regulation of Hsp70 chaperones.

### PES does not interact with Hsp70 in a specific way

**Figure 6:**
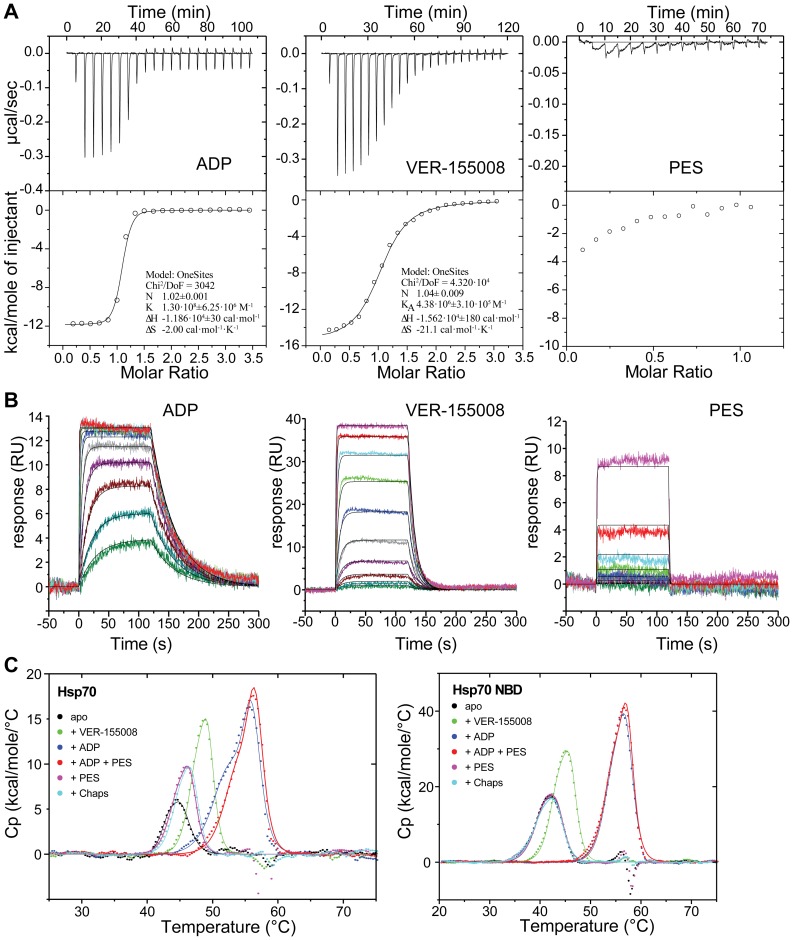
PES does not interact with a specific site in Hsp70. **A**, isothermal titration calorimetry of the interaction of ADP (left panel), VER-155008 (middle panel) and PES (right panel) to full-length human Hsp70. In the case of PES the titration was performed in the presence of ADP (see Figure S1). The determined K_d_ values were 7.7±0.4 nM (ADP) and 228±16 nM (VER-155008). **B**, Analysis of the interaction of ADP, VER-155008 and PES with Hsp70 by surface plasmon resonance spectroscopy. Hsp70 was immobilized on a CM5 chip (13.000 RU) followed by injections of ADP in the concentration range between 0.039 µM and 20 µM (left panel), of VER-155008 between 0.01 µM and 5 µM (middle panel) and PES between 0.39 µM and 200 µM (right panel). The experimental data (colored curves) were fitted to a 1∶1 binding model (black curves) resulting in slightly higher dissociations constants for ADP (K_d_ = 93 nM) and VER-155008 (K_d_ = 400 nM) than obtained by ITC since a running buffer without phosphate was used. For PES the data fitting delivered dubious results with an unrealistic maximal binding level and a K_d_ value in the triple digit millimolar range. **C**, Differential scanning calorimetry of C-terminally FLAG-tagged full-length Hsp70 (left panel) and the NBD of Hsp70 (right panel) in the absence of ligand (black), in the presence of 50 µM VER-155008 (green), 100 µM ADP (blue), 100 µM PES (magenta), 100 µM ADP+100 µM PES (red), and 100 µM CHAPS (3-[(3-cholamidopropyl)dimethylammonio]-1-propanesulfonate) (cyan). T_m_ and ΔH values are summarized in Table S4.

After analyzing all parameters of Hsp70 function, we were puzzled not to find any effect of PES on a single process of chaperone action, though refolding of heat denatured firefly luciferase was inhibited. Therefore, we performed isothermal titration calorimetry experiments (ITC) to analyze the binding behavior of PES to Hsp70 (Figure 6A). As a control we performed ITC experiments with ADP and VER-155008. ADP and VER-155008 titration resulted in perfectly sigmoidal binding curves representing a highly enthalpy driven binding interaction with affinities of 7.7±0.4 nM (ADP) and 228±16 nM (VER-155008), respectively. PES has been reported to bind specifically to the SBD of HSP70 and thus we performed titrations with Hsp70 in presence of excess ADP in order to block any unspecific binding to the nucleotide pocket. The titration yielded no sigmoidal curve in the concentration range used and the heat released with each injection was very small, providing no indication that PES would bind to Hsp70 in a specific manner. The same observation was made for titrations with the Hsp70-NBD under identical conditions. Interestingly, ITC experiments using the nucleotide-free Hsp70 revealed higher background signals for both protein species being more pronounced for the full-length Hsp70 compared to the Hsp70-NBD (Figure S1). This background signal is of unclear origin but cannot be attributed to a distinct stoichiometric binding. In addition, we analyzed binding of PES, and ADP and VER-155008 as control, to Hsp70 using surface plasmon resonance spectroscopy. Both, ADP and VER-155008, bound to Hsp70 following a 1∶1 Langmuir binding model (Figure 6B). In contrast, we could not obtain surface saturation for PES binding to Hsp70 within the concentration range tested (≤200 µM) indicating unspecific binding at higher micromolar concentrations or a dissociation constant of PES well above 100 µM, respectively (Figure 6B). Finally, we analyzed binding of PES, and ADP and VER-155008 as control, to Hsp70 using differential scanning calorimetry measuring the apparent melting temperature T_m_. Binding of ligands to the native state of a protein in the absence of binding to the denatured state will necessarily lead to stabilization of the complex and hence to an increase in T_m_. It should be noted that the T_m_ shift is a more qualitative measure for the complex stability at physiological temperatures as it is influenced in magnitude by multiple factors. Apo Hsp70 had a melting temperature of 44.6°C (Figure 6C). Addition of 50 µM VER-155008 or 100 µM ADP to Hsp70 increased the melting temperature by 4 and 11°C to 48.7°C and 55.7°C, respectively. This is in accordance to the relative affinities observed for these ligands by ITC and SPR. In the presence of 100 µM PES the melting temperature was also increased, albeit only slightly, by 1.4°C to 46°C. Interestingly, a similar increase of melting temperature was also achieved by the addition of the detergent 3-[(3-cholamidopropyl)dimethylammonio]-1-propanesulfonate (CHAPS). In the presence of ADP, PES increased the melting temperature by only 0.7°C to 56.3°C. No change of apparent T_m_ values was observed in presence of PES or CHAPS when only the NBD of Hsp70 was used (Figure 6C right panel and Table S4). All of these data cannot support a specific interaction of PES with Hsp70 under the conditions tested. If effects are observed, then only for full-length-HSP70 and with a magnitude that is also seen for unspecific interactions as for the detergent CHAPS.

## Discussion

In this study we demonstrate that down-regulation of the heat-inducible Hsp70 (HSPA1A/HSPA1B) to less than 10% of its cellular level does not suffice to challenge the different cancer cells tested. Similarly, down-regulation of the constitutively expressed Hsc70 (HSPA8) to the level achieved here (ca. 40% of its steady-state level) did not compromise viability of the cancer cells. A combined down-regulation of the constitutive Hsc70 (HSPA8) and prevention of up-regulation of the heat-inducible Hsp70 (HSPA1A, HSPA1B) was required to compromise cell viability. Furthermore, we analyzed the molecular mechanism of two proposed small molecule inhibitors of Hsp70 chaperones, one of which (VER-155008) was previously shown to bind to the NBD of Hsc70 and the other (PES) proposed to specifically interact with the SBD of heat-inducible Hsp70. Consistent with earlier observations for Hsc70, VER-155008 bound to the nucleotide binding site of both Hsc70 and Hsp70 and acted as an ATP-competitive inhibitor of ATPase and chaperone activity. By contrast, using biophysical methods we could not identify experimental evidence that PES would bind to any single binding site on Hsp70 in a specific and stoichiometric modality under our experimental conditions. Instead, PES may interact with low affinity with the SBD of Hsp70 in an unspecific, detergent-like way as demonstrated by DSC. Both compounds showed moderate inhibitory effects on the chaperone action of the constitutive Hsc70 and the heat inducible Hsp70.

Our data reporting on cancer cell viability following down-regulation of Hsp70 isoforms have significant implications concerning the strategies targeting Hsp70 for cancer therapy. Since down-regulation of both major cytoplasmic Hsp70s, Hsp70 (HSPA1A, HSPA1B) and Hsc70 (HAPA8) (Figure 1), was necessary to reduce viability of cancer cells from different lines, paralog specificity does not appear to be a desideratum for a clinically usable Hsp70 inhibitor. These findings are consistent with those of Powers and coworker [15] but contrast earlier data [44], although we have used similar cell lines. The major difference between our study and the earlier publication is the method used to down-regulate the Hsp70 levels: while the earlier study by Nylandsted and coworkers used adenoviral anti-sense constructs, we employed siRNA. Infection with an adenoviral vector may have sensitized the cells for Hsp70 depletion.

Our findings for VER-155008 are consistent with earlier observations [24], [41] and we could confirm that the compound is competing with ATP for binding to Hsp70. The crystal structure demonstrates that VER-155008 keeps the NBD in a conformation, which is about half way between the closed nucleotide bound state and the open conformation induced by the interaction with nucleotide exchange factors of the Bag-1 and Hsp110 families. As determined by differential scanning calorimetry, VER-155008 binding stabilizes Hsp70 but not to the extent achieved by nucleotides, most likely due to the prevention of the complete closure of the nucleotide binding cleft. The intrinsic ATPase activity of Hsp70 was inhibited with *K_i_* values of 10.9 and 2.9 µM in the absence or presence of the J-domain containing co-chaperone Hdj1, respectively (Figure 4). This difference is most likely caused by nucleotide release becoming rate limiting in the presence of Hdj1 [45]. Even more strikingly, we observed a slowdown of the association of fluorescently labeled nucleotide to Hsp70 by two orders of magnitude in the presence of VER-155008 (Figure 4, Table 1). As a functional consequence of this inhibition, the rates of ATP-induced opening of the SBD and acceleration of substrate release are reduced and thus refolding of the model substrate firefly luciferase is impaired (Figure 2, Figure 5). VER-155008 by itself did not trigger transmission of a signal to the SBD and we did not observe any influence of the compound on substrate binding.

Recently, PES, originally described as an inhibitor of p53-mediated apoptosis [25], was reported to promote cancer cell death by specifically inhibiting the heat-inducible Hsp70 and its interactions with co-chaperones without affecting the constitutively expressed Hsc70 [26]. In pull down experiments it was observed that the SBD of Hsp70 is required to detect an interaction between the chaperone and PES. Due to the lower sequence conservation of the SBD as compared to the NBD an inhibitory mechanism involving this domain could explain the proposed isoform specificity. As such an isoform specific inhibitor can help understanding the different roles of the two isoforms within the background of a living cell and can act as a specialized drug, we were eager to elucidate its mode of action. To our surprise PES inhibited, yet only slightly, the refolding of heat-denatured luciferase by both Hsp70 and Hsc70 (Figure 2), which is consistent with a more recent study, which detected also an interaction of biotinylated PES with Hsc70 [27]. As the interaction is supposed to be mediated *via* the SBD we put great efforts into analyzing substrate affinity and binding dynamics in the presence and absence of PES in detail (Table 1). Despite these efforts we were not able to detect any direct influence of PES on the interaction of Hsp70 with a peptide substrate. We also did not observe any influence of PES on the ATPase cycle of Hsp70. Finally, under our experimental conditions and with the concentrations used the compound did not reveal binding to a specific site within Hsp70 (Figure 6) but instead interacted with Hsp70 in an undefined, non-saturable and non-stoichiometric manner. For this interaction the SBD of Hsp70 was necessary (Figure 6C). How this interaction is able to inhibit the chaperone activity of Hsp70 is not clear. Based on the observation that deletion of the disordered C-terminal tail of the *Escherichia coli* Hsp70 homolog DnaK reduces slightly chaperone activity and cell viability under sever stress conditions it was proposed that the disordered C terminus of Hsp70s contains a weak substrate binding site. This site was not excluded as potential binding site for PES in our study. However, Hsp70 with a deleted C-terminal tail is pulled down with similar efficiency by biotinylated PES/avidin beads as wild type Hsp70 [46], excluding such a possibility. In contrast, single amino acid replacement variants of Hsp70 were shown recently to be resistant to pull-down by biotinylated PES/avidin beads [46]. These data suggest an interaction of PES with the helical lid. Interestingly, it was shown earlier that deletion of the helical lid in *E. coli* DnaK abrogates its ability to refold denatured firefly luciferase and compromises complementation of dnaK-deletion in vivo [10]. It is therefore possible that the helical lid contains additional low affinity substrate binding sites that are important for refolding. Unfortunately, such binding sites for substrate proteins have not been demonstrated directly so far and, to our knowledge, there is currently no assay available to test whether PES prevents such binding. Nevertheless, the existence of several such sites would explain the non-saturable low-affinity binding of PES detected in our study by surface plasmon resonance spectroscopy and by differential scanning calorimetry. Whether such a binding mode of PES is specific for Hsp70 and whether this is the mechanism by which PES acts *in vivo* remains to be shown, for example by rescuing PES-induced apoptosis and autophagy through expression of mutant but not wild type Hsp70.

Several other Hsp70 inhibitors of different classes have been described. Derivatives of spergualin, which are supposed to interact with the C-terminal EEVD motive, were reported to increase or decrease Hsp70 activity bringing the whole chaperone system out of balance [47]–[50]. Although the specificity and mode of action remain elusive, clinical trials against different cancer types were undertaken but without any result [51], [52]. The search for different scaffolds resulted in the identification of a series of dihydropyrimidines, which modulate Hsp40-mediated ATPase activity [53]. Although they exhibit weak activity and selectivity needs to be shown, some have anti-proliferative activity against cancer cell lines. A completely different class of inhibitors are proline-rich peptides, which specifically target the bacterial Hsp70 DnaK without harming mammalian Hsp70 [54], [55]. Structural analysis showed that the peptide binds to the SBD of DnaK generally in the same manner as a substrate does, but in addition to the competition for the substrate binding site also deregulates allosteric control [56], [57].

Modulation of the activity of Hsp70 chaperones offers a great possibility to influence protein homeostasis and cell survival making it a potential drug target. Due to the difficult environment of the ATP binding site [16] compounds influencing allosteric control of the chaperone cycle appear to be a promising direction to follow. However, further research is required to achieve the affinity and specificity required for the use of modulators of Hsp70 activity as a drug.

## Supporting Information

Figure S1 **PES does not interact with a specific site in Hsp70.** Isothermal titration calorimetry of the interaction of PES to nucleotide-free, full-length human Hsp70 (**A**), to nucleotide-free nucleotide binding domain of human Hsp70 (**B**), and to the nucleotide binding domain of Hsp70 in the presence of excess ADP (**C**).
(TIF)

Table S1 **Dual depletion of HSPA1 and A8 is also necessary to reduce colony formation of other cell lines.**
(PDF)

Table S2 **Dual depletion of HSPA1 and A8 is also necessary to reduce viability of other cell lines.**
(PDF)

Table S3 **Crystallographic data and refinement parameters.**
(PDF)

Table S4 **Apparent melting temperature for thermal unfolding of full-length human Hsp70 of the NBD of Hsp70 in the absence or presence of ligands.**
(PDF)
