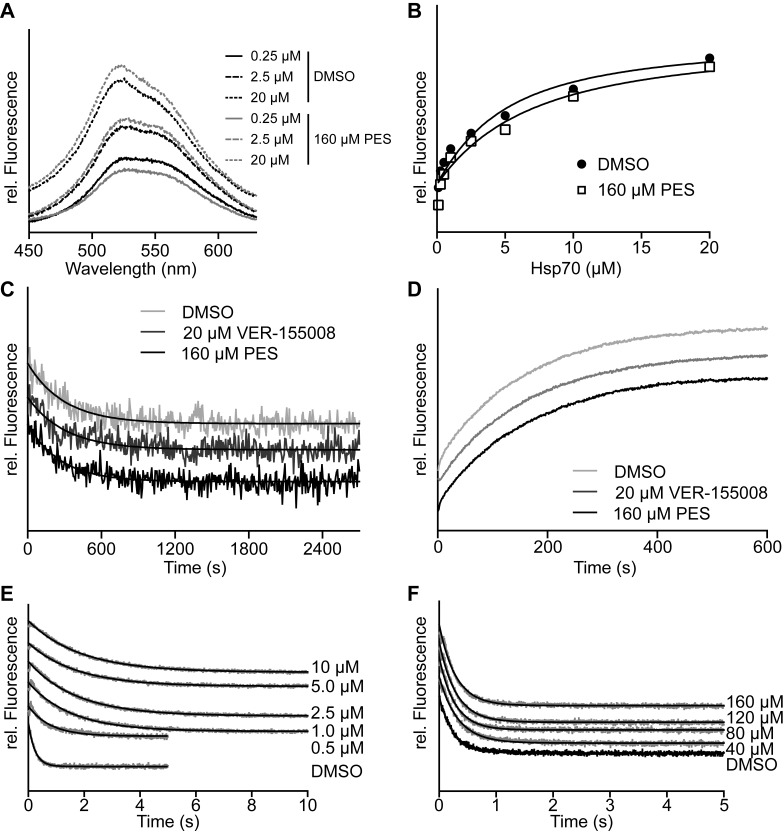# Correction: Functional Analysis of Hsp70 Inhibitors

**DOI:** 10.1371/annotation/5a7961d9-a7ea-4b10-9b48-5b106c405b02

**Published:** 2013-12-19

**Authors:** Rainer Schlecht, Sebastian R. Scholz, Heike Dahmen, Ansgar Wegener, Christian Sirrenberg, Djordje Musil, Joerg Bomke, Hans-Michael Eggenweiler, Matthias P. Mayer, Bernd Bukau

The image currently appearing as Figure 5 consists of Figures 4e and 4f, and belongs with the title and legend for Figure 4.

In addition, Figure 5 was incorrectly omitted from the manuscript. Please see the correct Figure 5 here: 

**Figure:**