# Supplementary figures and images for: Functional Analysis of Hsp70 Inhibitors
Source: PLoS One. 2013 Nov 12;8(11):e78443. doi: 10.1371/journal.pone.0078443 (PMC3827032; doi:10.1371/journal.pone.0078443)

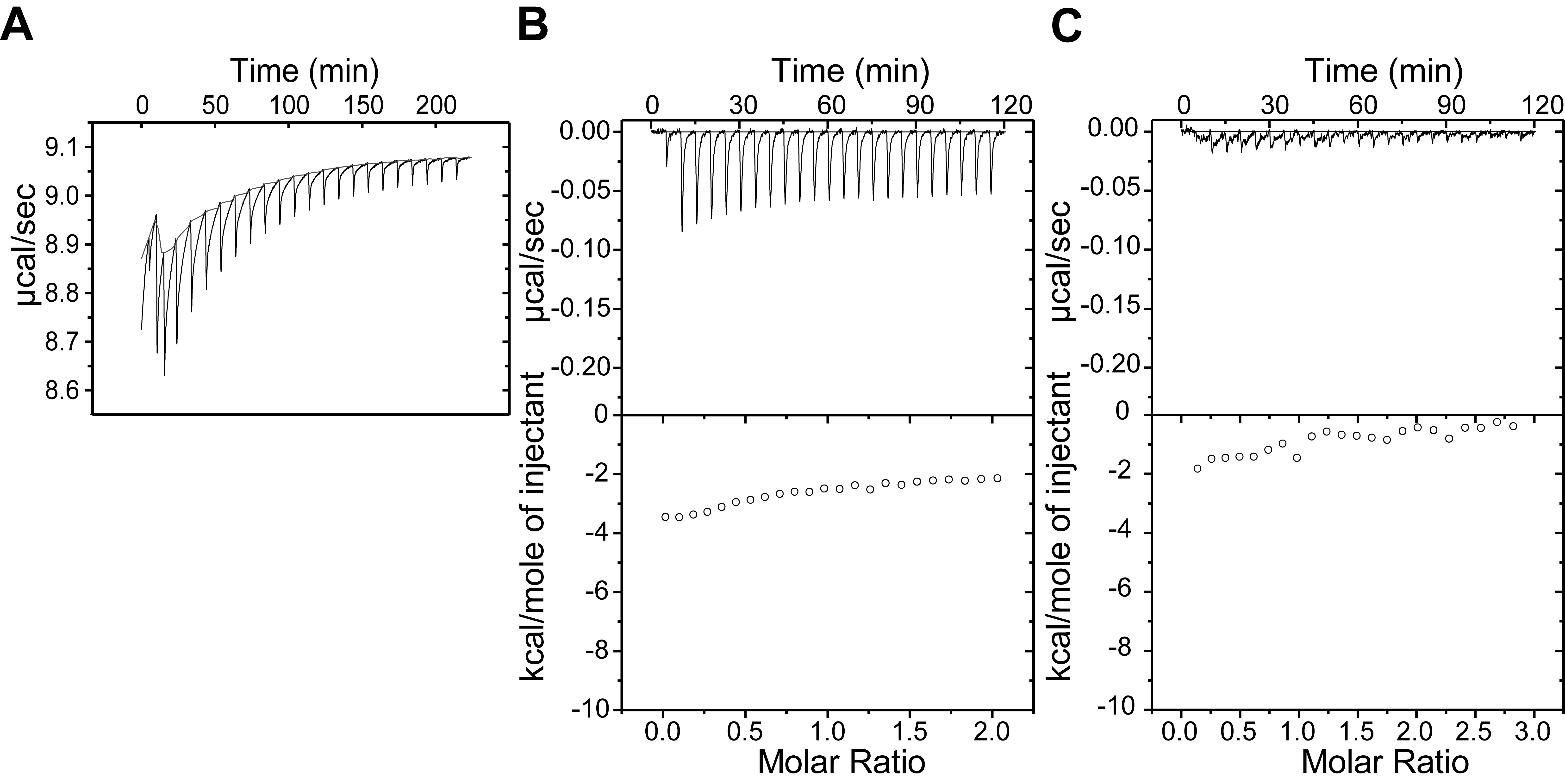

Supplement: Figure S1 — PES does not interact with a specific site in Hsp70. Isothermal titration calorimetry of the interaction of PES to nucleotide-free, full-length human Hsp70 (A), to nucleotide-free nucleotide binding domain of human Hsp70 (B), and to the nucleotide binding domain of Hsp70 in the presence of excess ADP (C). (TIF) [file pone.0078443.s001.tif]
