# Supplementary material for: Functional Analysis of Hsp70 Inhibitors
Source: PLoS One. 2013 Nov 12;8(11):e78443. doi: 10.1371/journal.pone.0078443 (PMC3827032; doi:10.1371/journal.pone.0078443)
Supplement: Table S1 — Dual depletion of HSPA1 and A8 is also necessary to reduce colony formation of other cell lines. (PDF) [file pone.0078443.s002.pdf]

Table S1: Dual depletion of HSPA1 and A8 is also necessary to reduce colony formation of other cell lines:

		Degree of reduction of colony formation by knocking down				
**Cell line**	**Cancer type**	**HSPA1**	**HSPA2**	**HSPA5**	**HSPA8**	**HSPA1+8**
**MDA-MB-468**	Breast cancer	+	-	-	-	++
**BT474**	Breast cancer	-	-	-	-	++
**HT29**	Colon cancer	-	-	-	-	++
**HCT 116**	Colon cancer	-	-	-	-	++

Cell lines BT474, HT29, HCT116, PC3, SKBr3 as well as MCF7 were obtained from ATCC; A2780 were obtained from ECAAC and MDA-MB-468 from DSMZ. Passages stored in the central cell bank at Merck Serono were tested for their identity by using STR (short tandem repeat) analysis. Cell lines were usually passaged for maximal 3-4 month and only passage number < 30 are used for the shown experiments. Cell lines were transfected with siRNAs targeting the indicated heat shock proteins. 24 h after transfection the cells were embedded in softagar. Growth of colonies was determined 6 days (in case of BT474, 9 days) after siRNA-transfection by measuring cell viability using Alamar Blue reagent (+++: 70-100% viability inhibition, ++: 40-70% viability inhibition, +: 10-40% viability inhibition, -: 0-10% viability inhibition in comparison to controls).
