# Supplementary material for: Functional Analysis of Hsp70 Inhibitors
Source: PLoS One. 2013 Nov 12;8(11):e78443. doi: 10.1371/journal.pone.0078443 (PMC3827032; doi:10.1371/journal.pone.0078443)
Supplement: Table S2 — Dual depletion of HSPA1 and A8 is also necessary to reduce viability of other cell lines. (PDF) [file pone.0078443.s003.pdf]

Table S2: Dual depletion of HSPA1 and A8 is also necessary to reduce viability of other cell lines

		Degree of reduction of viability by knocking down				
**Cell line**	**Cancer type**	**HSPA1**	**HSPA2**	**HSPA5**	**HSPA8**	**HSPA1+8**
**PC3**	Prostate cancer	-	-	+	-	++
**A2780**	Ovarian cancer	-	-	-	-	-
**SKBr3**	Breast cancer	-	-	-	-	+
**MCF7**	Breast cancer	+	-	++	-	++

Cell lines were transfected with siRNAs targeting the indicated heat shock proteins. Cell viability was determined 6 days after transfection by using Alamar Blue reagent (+++: 70-100% viability reduction, ++: 40-70% viability reduction, +: 10-40% viability reduction, -: 0-10% viability reduction in comparison to controls).
