# Supplementary material for: Functional Analysis of Hsp70 Inhibitors
Source: PLoS One. 2013 Nov 12;8(11):e78443. doi: 10.1371/journal.pone.0078443 (PMC3827032; doi:10.1371/journal.pone.0078443)
Supplement: Table S3 — Crystallographic data and refinement parameters. (PDF) [file pone.0078443.s004.pdf]

Table S3: Crystallographic data and refinement parameters.

Data-collection details	
Space group	$P2_1$
Unit-Cell parameters [Å, °]	a=58.68, b= 53.97, c= 70.80;
	α = 90.0 β = 101.1 γ = 90.0
Resolution [Å]	2.6
Data collection wavelength [Å]	1.00000
Number of measurements	36 077
Number of unique reflections	12 776
Completeness[%] [a]	92.3 (82.0)
R_{merge} [%]	9.0 (36.4)
$<I/\sigma(I)>$[a]	10.1 (2.5)
Refinement	
Nr. of molecules / asymmetric unit	1
R_{work}/R_{free}[b]	0.167 / 0.239
Rmsd bond length [Å]	0.010
Rmsd bond angle [°]	1.1
Average B-factor [$Å^2$]	
Protein	33.8
Ligand	27.8
Water	30.3
All atoms	33.6
Number of atoms	
Protein atoms	2938
Ligand	38
Water	121
All atoms	3097
Ramachandran	
G-factor	0.08
Core [%]	91.6
Allowed [%]	8.4
Generously allowed [%]	0.0
Dissallowed [%]	0.0
