# Supplementary material for: Functional Analysis of Hsp70 Inhibitors
Source: PLoS One. 2013 Nov 12;8(11):e78443. doi: 10.1371/journal.pone.0078443 (PMC3827032; doi:10.1371/journal.pone.0078443)
Supplement: Table S4 — Apparent melting temperature for thermal unfolding of full-length human Hsp70 of the NBD of Hsp70 in the absence or presence of ligands. (PDF) [file pone.0078443.s005.pdf]

Table S4: Apparent melting temperature for thermal unfolding of full-length human Hsp70 or the NBD of Hsp70 in the absence or presence of ligands

	Full-length Hsp70 appT$_m$ (°C)	Hsp70 NBD appT$_m$ (°C)
apo	44.6	42.2
100 μM ADP	55.7	56.5
50 μM VER-155008	48.7	45.3
100 μM PES	46.0	42.2
100 μM PES + 100 μM ADP	56.3	56.7
100 μM Chaps	46.4	42.2
